# Closing the RCT Gap—A Large Meta-Analysis on the Role of Surgery in Stage I–III Small Cell Lung Cancer Patients

**DOI:** 10.3390/cancers16112078

**Published:** 2024-05-30

**Authors:** Fabian Doerr, Sebastian Stange, Sophie Salamon, Konstantinos Grapatsas, Natalie Baldes, Maximilian Michel, Hruy Menghesha, Georg Schlachtenberger, Matthias B. Heldwein, Lars Hagmeyer, Jürgen Wolf, Eric D. Roessner, Thorsten Wahlers, Martin Schuler, Khosro Hekmat, Servet Bölükbas

**Affiliations:** 1 Department of Thoracic Surgery, West German Cancer Center, University Medical Center Essen-Ruhrlandklinik, University Duisburg-Essen, 45239 Essen, Germany; 2 Department of Thoracic Surgery, Regiomed-Klinikum Coburg GmbH, 96450 Coburg, Germany; 3 Clinic for Oral Surgery, 40879 Ratingen, Germany; 4 Institute of Zoology, Faculty of Mathematics and Natural Sciences, University of Cologne, 50674 Cologne, Germany; 5 Department of Thoracic Surgery, Helios Clinic Bonn/Rhein-Sieg, 53123 Bonn, Germany; 6 Division of Thoracic Surgery, Department of General, Thoracic and Vascular Surgery, Bonn University Hospital, 53127 Bonn, Germany; 7 Department of Cardiothoracic Surgery, University Hospital of Cologne, University of Cologne, 50937 Cologne, Germany; 8 Clinic for Pneumology and Allergology, Bethanien Hospital GmbH, 42699 Solingen, Germany; 9 Lung Cancer Group Cologne, Department I of Internal Medicine, University Hospital Cologne, University of Cologne, 50937 Cologne, Germany; 10 Interdisciplinary Thoracic Center, Division of Thoracic Surgery, University Hospital of Mainz, University of Mainz, 55131 Mainz, Germany; 11 Department of Medical Oncology, West German Cancer Center, University Medical Center Essen, University Duisburg-Essen, 45147 Essen, Germany; 12 National Center for Tumor Diseases (NCT) West, Campus Essen, 45147 Essen, Germany

**Keywords:** small cell lung cancer, surgery, meta-analysis, 5-year survival

## Abstract

**Simple Summary:**

Despite guidelines recommending surgery as part of treatment for stage I small cell lung cancer (SCLC), its application remains inconsistent, and its role in stages II and III is under debate. In absence of current randomized control trials this meta-analysis compared surgery to non-surgical treatment for stages I to III SCLC. After a systematic review, ten studies with a total of 95,323 patients were analyzed. The analysis found no significant differences in patient characteristics between the surgery and non-surgery groups. The 5-year survival rate for resected patients was significantly higher compared to non-surgically treated patients. This finding was valid also for patients in stages II and III. Surgery might significantly improve survival in SCLC patients and should be considered in treatment planning even in higher stages.

**Abstract:**

Introduction: Despite clear guideline recommendations, surgery is not consistently carried out as part of multimodal therapy in stage I small cell lung cancer (SCLC) patients. The role of surgery in stages II and III is even more controversial. In the absence of current randomized control trials (RCT), we performed a meta-analysis comparing surgery versus non-surgical treatment in stage I to III SCLC patients. Methods: A systematic review of the literature was conducted on 1 July 2023, focusing on studies pertaining to the impact of surgery on small cell lung cancer (SCLC). These studies were evaluated using the ROBINS-I tool. Statistical analyses, including I² tests, Q-statistics, DerSimonian-Laird tests, and Egger regression, were performed to assess the data. In addition, 5-year survival rates were analyzed. The meta-analysis was conducted according to PRISMA standards. Results: Among the 6826 records identified, 10 original studies encompassing a collective cohort of 95,323 patients were incorporated into this meta-analysis. Heterogeneity was observed across the included studies, with no discernible indication of publication bias. Analysis of patient characteristics revealed no significant differences between the two groups (*p*-value > 0.05). The 5-year survival rates in a combined analysis of patients in stages I–III were 39.6 ± 15.3% for the ‘surgery group’ and 16.7 ± 12.7% for the ‘non-surgery group’ (*p*-value < 0.0001). SCLC patients in stages II and III treated outside the guideline with surgery had a significantly better 5-year survival compared to non-surgery controls (36.3 ± 20.2% vs. 20.2 ± 17.0%; *p*-value = 0.043). Conclusions: In the absence of current RCTs, this meta-analysis provides robust suggestions that surgery might significantly improve survival in all SCLC stages. Non-surgical therapy could lead to a shortening of life. The feasibility of surgery in non-metastatic SCLC should always be evaluated as part of a multimodal treatment.

## 1. Introduction

Small cell lung cancer (SCLC) accounts for approximately 10–15% of all malignant lung tumors. Because of its malignant nature, characterized by rapid growth of the primary lesion and early dissemination to mediastinal lymph nodes or distant organs, it ranks as the fifth most common cause of cancer-related mortality [1]. Since SCLC is a strongly nicotine-associated tumor, it is associated with numerous mutations, such as the loss of function of the tumor suppressor genes Tp53 and RB1 [2]. Hence, the prognosis of SCLC is generally poor, and median survival in stage III is below a year [3]. 

Guidelines on the treatment of SCLC are ambivalent and suggest either surgery or non-surgery treatment for stage I SCLC [4,5]. The leading recommendation for stage II and III SCLC patients is not surgery but instead radio-/chemotherapy [4,5]. Furthermore, surgery alone is considered to be an inadequate therapeutic approach in SCLC. Even though surgery improves the long-term prognosis of stage I SCLC patients [6], the number of operations performed in the past decade has stagnated [7]. In recent years, less than 10% of potentially resectable patients underwent surgery [8]. In contrast, several groups have recently published (1) a positive long-term effect after surgery in SCLC patients in T3- or T4-stages [6,9], and (2) promising results after surgery in SCLC patients with positive lymph node involvement [9,10]. These studies suggest that current guidelines should be stronger in the integration of surgical therapy for early-stage SCLC. 

At the time of writing this, only three randomized control trials (RCTs) were completed on the role of surgery in SCLC [11,12,13]. However, these trials are older and include a total of not more than 330 patients, almost half of whom were recruited before 1965. In the absence of modern staging technology and up-to date surgical techniques, surgery was found to be inferior to non-surgery treatment. Undoubtedly, a modern RCT would guarantee the best level of evidence [14]. Sadly, due to the rarity of resectable patients, conducting an RCT to evaluate the role of surgery in SCLC is complicated and may not be feasible in a single-site study [6].

The objective of this study was to evaluate whether recent evidence is indicative of or against the use of surgery in early-stage SCLC. We assessed the role of surgery in stage I, stage II, and stage III SCLC by comparing the 5-year survival rates of patients who underwent surgery compared to patients with no surgical treatment. Our meta-analysis, comprising almost 100,000 SCLC patients from 10 retrospective studies, shows a significant impact of surgery on 5-year survival.

## 2. Material and Methods

### 2.1. Study Inclusion and Exclusion Criteria

This systematic review adhered to the ‘Preferred Reporting Items for Systematic Reviews and Meta-Analyses’ (PRISMA) guidelines [15]. Databases were searched for studies published from 2004 onwards, focusing on investigating the impact of surgery in stages I, II, and III of SCLC. Considering the evolution in lung cancer staging over time, inclusion criteria were limited to studies that used modern staging tools such as computed tomography [16].

**Figure 1:**
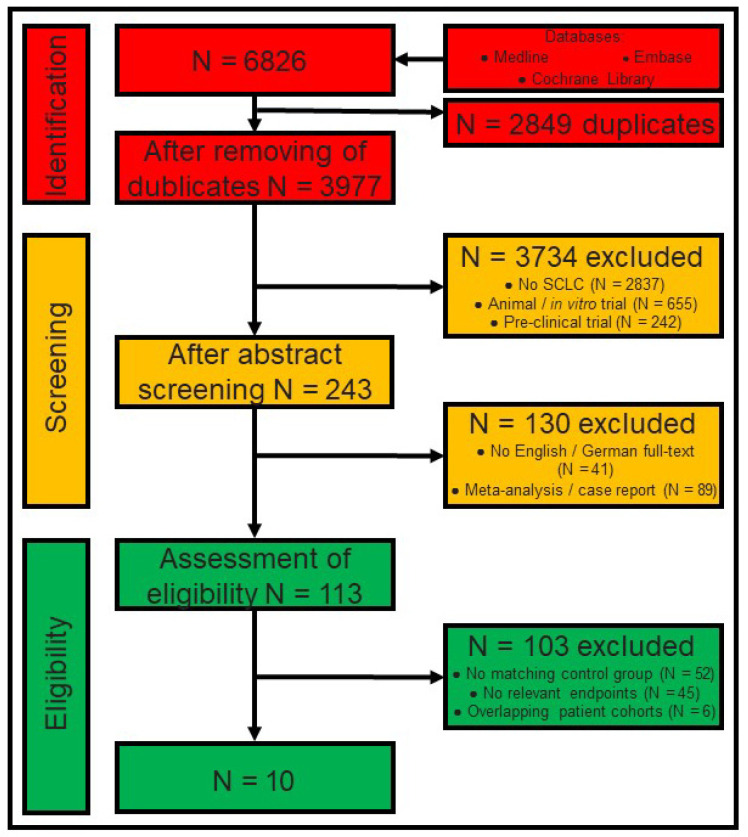
Flowchart of the literature research: The diagram illustrates the ‘identification’ (in red) of studies following the literature research across three databases. Subsequently, a ‘screening’ process (in orange) was conducted to identify relevant articles, which were ultimately deemed ‘eligible’ (in green) for inclusion in the meta-analysis. The colored boxes in the middle depict the number of articles at each stage of assessment. On the right, colored boxes display the number of excluded articles along with the reasons for exclusion. Abbreviation: SCLC: small cell lung cancer.

Hence, patient recruitment in the original study had to be within the last 35 years to avoid bias due to inaccurate staging. We identified and analyzed studies in accordance with the criteria listed in Figure 1.

The ‘non-surgery group’ was delineated as comprising patients who solely underwent radiotherapy or chemotherapy treatment. Conversely, patients who received surgical intervention alone or in combination with neoadjuvant or adjuvant therapy were categorized as the ‘surgery group’. The definitions of clinical endpoints were adopted directly from the primary publications. Although disease-free survival (DFS) is superior to overall survival (OS) as a primary endpoint when considering clinical factors such as patient fitness, our meta-analysis is based on OS data because DFS was not available in all included studies.

### 2.2. Search Strategy

Two authors (FD and SeS) performed an independent literature search on 1 July 2023, according to predefined standards [17,18] to guarantee that no patient is included multiple times in the meta-analysis despite compiling cohorts from similar data sources. Figure 1 provides a detailed flowchart of the search strategy.

### 2.3. Data Extraction, Quality, and Bias Assessment

All pertinent data, encompassing demographic information and the primary endpoint of interest, were extracted from the original studies. In instances where information was missing, the first or senior author of the respective study was contacted for clarification. Assessment of study quality and risk of bias was conducted by two independent investigators (FD and SeS) using the ROBINS-I tool (version for cohort-type studies; 19 September 2016) [19]. To avoid a staging bias, all studies were evaluated regarding predefined staging conditions [17,18].

### 2.4. Statistical Analysis

The implementation of the statistical analyses was described in detail in previous publications [17,18,20,21].

## 3. Results

### 3.1. Literature Search

**Figure 2:**
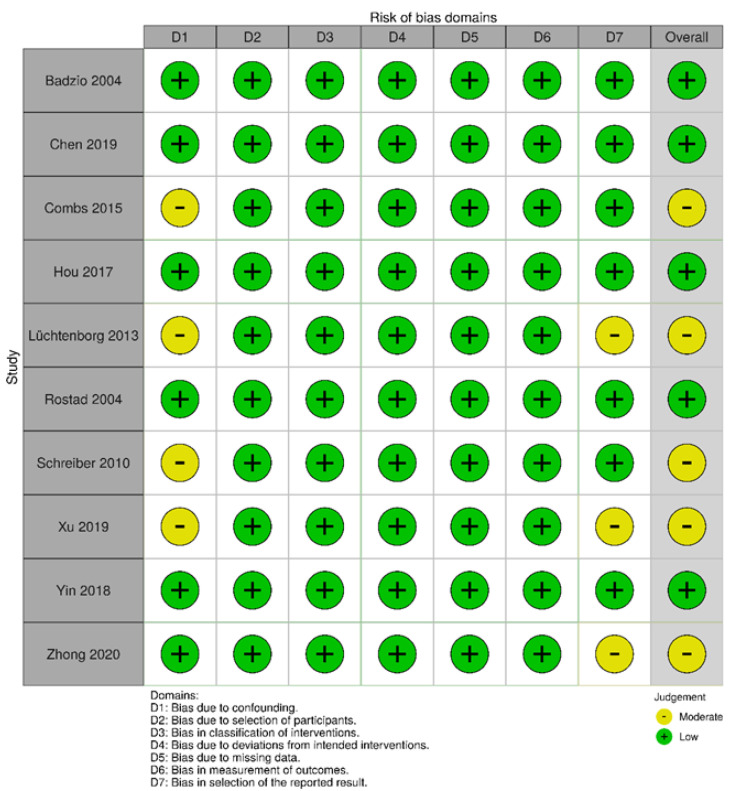
Overall risk of bias according to ROBINS-I [8,9,22,23,24,25,26,27,28,29].

From an initial pool of 6826 papers identified through systematic literature research, 10 studies were incorporated into the meta-analysis (Figure 1). According to the ROBINS-I assessment, the overall risk of bias in the included studies was deemed low to moderate (Figure 2). The publication dates of these studies spanned from 2004 to 2020 (Table 1). Notably, Lüchtenborg et al. conducted the study with the longest patient recruitment period, spanning 21 years from 1989 to 2009 [22]. Conversely, Yin et al. conducted one of the most recent studies, with a recruitment period of six years from 2010 to 2015 [23]. All the included studies were retrospective in nature. Among them, five studies used national data registries, while the remaining five studies were based on single-center data. The study by Hou et al., published in 2017, boasted the largest dataset, comprising 208 patients [24]. Three studies used a pair-match analysis (Table 1) [23,25,26]. Despite meeting the inclusion criteria, we did not consider six studies for this meta-analysis. These studies cover overlapping patient cohorts from similar data sources (SEER and NCDB). To prevent patients from being included multiple times, we only considered the studies with the most complete dataset. The data from the six excluded studies are presented in the lower segment of Table 1.

### 3.2. Patient Details

A total of 95,323 patients were included in this meta-analysis. Of these, 3569 patients were distributed to the ‘surgery group’ and 91,754 to the ‘non-surgery group’. Patients’ mean age was 64.2 ± 4.7 years, and 57.4 ± 15.2% of all patients were male. Patient characteristics did not significantly (*p*-value > 0.05) differ between the ‘surgery group’ and ‘non-surgery group’ (Table 2). The combined stage I–III analysis was executed on all patients. In contrast, the analysis of patients in stages II and III is based on 40,705 patients (surgery: n = 1059; non-surgery: n = 39,646).

### 3.3. 5-Year Survival Analysis in Stages I–III

**Figure 3:**
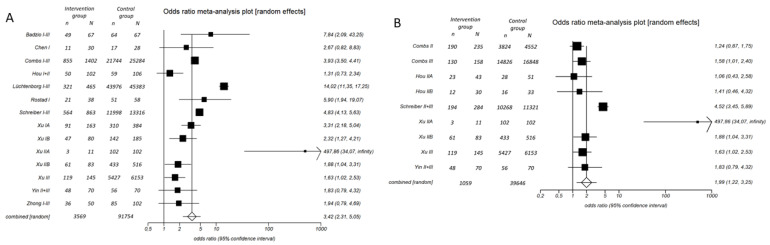
Odds ratio meta-analysis plot in stages I–III (**A**) and II–III (**B**). The figure depicts the results of the meta-analysis for stages I–III (**A**) and stages II–III (**B**). The names listed on the left represent the first authors of the original studies. Studies may appear multiple times if different stages of small cell lung cancer (SCLC) are included in one analysis. In the intervention group, patients underwent surgery, while in the control group, they did not. ‘n’ denotes the number of individuals in the group with the outcome, while ‘N’ indicates the total number in the group. An odds ratio greater than 1 signifies evidence favoring the superiority of surgery. The size of the squares corresponds to the sample size. On the right, numbers display the odds ratio and 95% confidence interval for each study [8,9,22,23,24,25,26,27,28,29].

The 5-year survival analysis for stages I–III exhibited a significant discrepancy in the Q-statistic (*p*-value < 0.0001), with the I² test indicating 93.9% inconsistency (95% CI: 91.8–95.2%). These findings provide compelling evidence of substantial statistical heterogeneity among the included studies. Consequently, the DerSimonian and Laird random-effects model was employed. The pooled odds ratio was calculated at 3.4 (95% CI: 2.3–5.1), with a Chi² value of 38.1 (*p*-value < 0.0001). This outcome suggests a significant enhancement in the 5-year survival endpoint for patients in the ‘surgery group’ compared to control patients (Figure 3A). Egger’s weighted regression statistic yielded a *p*-value of 0.4574, indicating the absence of publication bias. A post-hoc power analysis indicated sufficient power for this analysis and its findings (effect size d: 1.60; power (1-β): 1.0; alpha error: 0.05). Consequently, surgical intervention significantly improved 5-year survival (*p*-value < 0.0001). The 5-year survival rates were reported as 39.6 ± 15.3% in the ‘surgery group’ versus 16.7 ± 12.7% in the ‘non-surgery group’ (Table 3 and Figure 3A). The absolute risk reduction (ARR) was calculated as 22.9 ± 17.2%, and the relative risk reduction (RRR) was determined as 26.7 ± 16.9%. For one patient to reach a 5-year survival in stage I–III SCLC, seven patients need to be resected (NNT: 6.6) (Table 3).

### 3.4. 5-Year Survival Analysis in Stage II and III Combined

The significant improvement in 5-year survival, which was shown above, might be driven by the stage I sub-population. Therefore, we conducted a specific analysis of 5-year survival rates among patients diagnosed with stage II and III SCLC. The Q-statistic for the 5-year survival endpoint yielded significance (*p*-value < 0.0001), with the I² test indicating 87.2% inconsistency (95% CI: 77.5–91.6%), thus reaffirming significant heterogeneity among the included studies. Using the DerSimonian and Laird random-effects model, similar to the previous analysis, we determined a pooled odds ratio of 2.0 (95% CI: 1.2–3.2), with a Chi² value of 7.7 (*p*-value = 0.0056). These findings suggest a noteworthy enhancement in 5-year survival among stage II–III SCLC patients following tumor resection (Figure 3B). Egger’s weighted regression statistic did not reveal significant publication bias (*p*-value = 0.7726), and a subsequent post-hoc power analysis demonstrated robust statistical power (effect size d: 1.79; power (1 − β): 1.0; alpha error: 0.05). Therefore, surgical intervention was associated with improved 5-year survival rates (*p*-value = 0.043). The 5-year survival rates were documented as 36.3 ± 20.2% in the ‘surgery group’ compared to 20.2 ± 17.0% in the ‘non-surgery group’ (Table 3 and Figure 3B). The ARR was calculated as 16.1 ± 22.9%, while the RRR was determined as 18.3 ± 22.4%. For one patient to reach a 5-year survival in stage II or III SCLC, 20 patients need to be surgically treated (NNT: 19.2) (Table 3).

## 4. Discussion

### 4.1. SCLC Prognosis Has Not Improved in Decades

In recent years, treatment modalities for non-small cell lung cancer (NSCLC) have undergone a revolution, resulting in markedly enhanced prognoses, even in the advanced stages of the disease [36]. In contrast, while in the past half century, more than 40 phase 3 trials on SCLC were finalized, the overall 5-year survival rates for small cell lung cancer (SCLC) persistently linger below 10% [36,37,38,39]. Since Topotecan almost 25 years ago, nivolumab was the first drug approved by the FDA in 2018 [40]. Presently, with an enhanced comprehension of the biology of SCLC, a plethora of therapeutic options, including targeted therapies and immunotherapies, emerge as promising contenders to potentially resolve this dilemma [37]. Studies in animal models have shown various promising candidates [36], and we recently showed in mouse models that SCLC displays an actionable dependence on ATR/CHK1-mediated cell cycle checkpoints [2]. Nonetheless, it is imperative to delve deeper into potential cellular and molecular mechanisms, necessitating further investigation. Translational research on SCLC faces challenges due to restricted access to human tumor tissue; thus, intensification of efforts in this domain is warranted. As research into targeted therapy options for SCLC is still in its infancy, an increased focus on translational research is crucial [36].

### 4.2. Surgery Is Underused in Early-Stage SCLC

In accordance with guidelines, surgery is recommended over non-surgical therapies for stage I node-negative T1/2 small cell lung cancer (SCLC) patients due to its favorable impact on the long-term prognosis [4,5,39]. However, there has been stagnation in the number of surgical procedures performed over the past decade [7], with only 10% of all potentially resectable patients [8] and 20% to 30% of stage I patients undergoing surgery [6,7,8,34]. This indicates a significant underutilization of surgery in SCLC cases [35]. Presently, only a third of early-stage SCLC patients are being referred for surgical consultation [41], and more than two-thirds of stage I patients are not offered surgery despite the absence of clear contraindications [39]. Even if patients with contraindications to surgery are excluded, the number of patients treated surgically is significantly lower than expected [7].

It appears that treatment of SCLC patients is clearly inconsistent and even varies within one region [7,39]. In an attempt to deliver the highest quality care, Wakeam et al. suggest a multidisciplinary discussion for all patients in stages I to III of SCLC [39]. It was shown that these meetings have a positive impact on the treatment of lung cancer patients, especially when surgeons play a key role [42]. This approach is important for enhancing the use of surgery in a guideline-concordant manner [42]. However—to close the gap—internists, oncologists, and pneumologists should consider surgery for SCLC patients [7].

The question that arises in this context is—why is surgery so underused as a treatment option in early-stage SCLC? 

### 4.3. The Case against Surgery in SCLC—Are Pre 90s RCTs All She Wrote?

Contemporary surgical protocols predominantly derive from three randomized controlled trials (RCTs) conducted by Fox et al. [11], Lad et al. [12], and Liao et al. [13]. These trials represent a robust level of evidence [14], yet they demonstrate minimal to negligible improvement in surgical interventions compared to alternative treatments in SCLC. Although a recent Cochrane review has highlighted challenges in interpreting these studies within the context of current standards, the authors persist in advocating against surgery for early-stage SCLC [43].

In contrast, we contend that assessing the role of surgery in SCLC solely based on these three RCTs may not be justified in the present context. The overall patient cohort is relatively small, and the staging procedures employed resulted in the inclusion of participants with advanced SCLC stages, rendering them unsuitable candidates for surgery by today’s standards. Moreover, the treatment protocols used in these RCTs exhibit heterogeneity and fail to meet contemporary surgical standards and recommendations, particularly with regards to the notable frequency of pneumonectomies. Furthermore, post-surgical outcomes are subject to bias due to a significant proportion of incomplete or non-executed resections. Despite these limitations, Lad et al. and Liao et al. do not report the significant inferiority of surgical intervention [12,13].

In 1973, Fox et al. published an RCT on the long-term survival of 144 patients that were recruited between 1962 and 1964 [11]. In this study, 71 patients were randomized into a surgery group and 73 patients into a radiotherapy group. Most participants enrolled in this study had advanced disease. The authors achieved complete resection by performing a pneumonectomy in 48% of surgical cases. Here, 34% of patients in the surgery arm underwent an explorative thoracotomy. Despite being randomized to surgery, 18% of the patients did not undergo any surgical therapy. The mean survival after surgery was only 199 days, whereas patients after radiotherapy survived 300 days (*p*-value = 0.04). Moreover, 1-year survival was 21% after surgery and 22% after radiotherapy. The 5-year and 10-year survival rates favored radiotherapy (1% versus 4% and 0% versus 4%, respectively). The inferiority of surgery might be due to the high rate of pneumonectomy and explorative thoracotomy rather than the beneficial effect of administered radiotherapy.

Lad et al. recruited patients between 1983 and 1989 [12]. The majority had bulky nodal disease. All participants underwent induction chemotherapy (cyclophosphamide, doxorubicin, and vincristine). Objective responders were subsequently randomized either to surgery (N = 70) followed by chest and whole brain radiation or to chest and whole brain radiation alone (N = 76). In this study, only 77% of patients in the surgery arm were completely resected (R0). In 6% of the cases, an incomplete resection was performed. In addition, 17% of the patients in the surgery group were considered unresectable after thoracotomy. Lad et al. found no significant survival difference between the two treatment groups. The median survival after surgery was 15.4 months versus 18.6 months without surgical treatment (*p*-value = 0.78).

The recruitment period of the RCT by Liao et al. ranged from January 1990 until December 1991 [13]. After giving induction chemotherapy (ifosfamide, adriamycin, and vincristine) to all participants, the authors randomized a cohort of 40 patients to surgery or radiotherapy. The randomized treatment was followed by chemotherapy for both groups. Liao et al. provide no information on the surgery performed. Neither surgical techniques nor resection margins are reported. Due to the small number of participants, the major finding of this RCT was statistically not significant, but higher survival rates were found after surgery compared to radiotherapy treatment (1-year: 79% versus 63%; 2-year: 52% versus 18%; 3-year: 24% versus 18%; *p*-value = 0.12). While the study by Liao et al. is the most recent, the power of the analysis is not overwhelming.

We consequently believe that there is a significant ‘RCT gap’ specifically in non-metastatic SCLC. However, an adequately powered RCT to evaluate the role of surgery in SCLC might never be completed [6], particularly as these early stages of SCLC only account for approximately 5% of all patients.

### 4.4. Survival Is Stage-Dependent: The Retrospective Papers

Several retrospective studies provide evidence of stage-dependent survival. Therefore, it is unlikely that the outcomes of our meta-analysis would undergo significant alterations by incorporating the six studies that were excluded owing to the overlapping patient cohorts sourced from similar data pools. Wei et al. documented a 19.9% 5-year survival advantage for surgical candidates across stages I to III using the SEER database [33]. The SEER database was further sourced for patients in stages I and II by Jin et al. [30], Varlotto et al. [32], Weksler et al. [34], and Lin et al. [31]. The 5-year survival benefit after surgery ranged from 16.8% to 30.2% in these studies. A survival benefit after surgical resection of SCLC compared to no surgical treatment was already reported over 20 years ago [44,45,46]. Recently, several authors analyzed the survival benefit according to clinical SCLC stages and even subdivided the stages to get more precise results [27].

Takenaka et al. managed to demonstrate a significant 5-year survival benefit for surgically treated patients compared to non-surgical treatment in stage I SCLC (62% versus 25%, *p*-value < 0.001) [47]. Furthermore, resected patients in stage II and III SCLC had a 5-year survival benefit in a propensity score-matched sub-group analysis [47]. Takenaka et al. have enrolled patients since 1974 and were consequently not considered for this meta-analysis. Another single-center study that did not meet inclusion criteria due to the low number of total patients (<125 individuals) was published by Gu et al. The authors report a 5-year survival benefit for surgically treated stage I–IIIB patients (31.1% versus 23.0%) [48].

Combs et al. provided evidence for a stage-specific 5-year survival benefit after surgery (stage I: 51% survival, stage II: 25%, and stage III: 18%) [8]. In 2012, Weksler et al. compared the median survival of surgically and non-surgically treated patients in the early SCLC stages. Lung resection significantly improved the patients’ survival (stage I: 38 months versus 16 months, *p*-value < 0.001; stage II: 25 months versus 14 months, *p*-value < 0.001) [34]. Rostad et al. showed a stage-dependent benefit of surgical treatment utilizing a 5-year survival endpoint in their combined stage IA and IB study, with 44.9% versus 11.3% survival, respectively [28]. Finally, Zhang et al. postulate a significant survival benefit of surgery for stage III SCLC patients [49].

Despite these robust findings, the role of surgery in the treatment of limited-disease SCLC is still under debate. Several recent authors argue for the necessity of a prospective randomized trial to finally answer this question [23,28,34,44,45,47]. As we already mentioned, it is unclear or unlikely whether a new RCT is on the horizon to date [7]. Instead, we turned to the utility of a meta-analysis of all retrospective studies to clarify the controversy for or against surgery in early and advanced-stage SCLC.

### 4.5. Can This Meta-Analysis Strengthen the Role of Surgery in SCLC?

Including almost 100,000 patients in a meta-analysis leads to robust data, especially when all the data sources are of good quality and meet the requirements for heterogeneity. The included studies showed no evidence of publication bias or high variance in baseline characteristics. This meta-analysis demonstrates a significant (*p*-value < 0.0001) 5-year survival benefit after surgery for patients up to stage III. Their 5-year survival advantage reached 22.9% (surgery: 39.6% versus non-surgery: 16.7%). The number needed to treat was 6.6, and every seventh SCLC patient up to stage III treated with surgery reaches 60 months of survival. In a second analysis, we only looked at SCLC stages II and III, where an operation is not recommended by any international guideline today. These patients also benefit significantly (*p*-value = 0.043) from surgery, and their 5-year survival rate nearly doubles (surgery: 36.3% versus non-surgery: 20.2%).

Some of the studies included in this meta-analysis deliver remarkable results. The 5-year survival benefit of stage I–III SCLC patients after surgery varied between 11.3% [29] and 27.9% in a study by Lüchtenborg et al. [22]. The three studies that examined matched patient groups showed the clear superiority of surgery over non-surgical therapy. Chen et al. analyzed stage I only (62.3% versus 40.1%) and Badzio et al. stage I–III (27.0% versus 4.0%) patients [25,26]. Even Yin et al., who analyzed stage II and III patients only, showed a superiority of surgical therapy (31.7% versus 20.0%) [23].

This meta-analysis strengthens the role of surgery not only in SCLC stage I but also up to stage III.

We could summarize that surgery improves the long-term survival of non-metastatic SCLC patients. The 5-year survival rates after surgery were significantly better compared to non-surgery in both analyses. We provided evidence that SCLC patients in non-metastatic stages should be presented to an interdisciplinary tumor board and resected whenever possible. We believe this meta-analysis will significantly strengthen the role of surgery in SCLC.

## Figures and Tables

**Table 1:** Overview of all original studies.

Author	Year	Period	Origin	Comment	5-y Surv. (%)		Patients (N)		
					Surg.	NS	All	Surg.	NS
Badzio [25]	2004	1984–1996	SC	St. I–III, PM	27.0	4.0	134	67 ^§P^	67 ^^^
Chen [26]	2019	2000–2016	SC	St. I, PM	62.3	40.1	58	30 ^§^	28 ^°P^
Combs [8]	2015	1998–2006	NCDB	St. I–III	39.0	14.0	26,686	1402 ^$-^	25,284 ^^^
Hou [24]	2017	2005–2010	SC	St. I + II	50.9	44.3	208	102 ^$+^	106 ^^^
Lüchtenb. [22]	2014	1989–2009	NCDR	St. I–III	31.0	3.1	45,848	465 ^0^	45,383 ^n^
Rostad [28]	2004	1993–1999	CR	St. I	44.9	11.3	96	38 ^§+^	58 ^^^
Schreiber [9]	2010	1988–2002	SEER	St. I–III	34.6	9.9	14,179	863 ^%^	13,316 ^n^
Xu [27]	2019	2010–2015	SEER	St. IA	43.9	19.2	547	163 ^%+^	384 ^^^
Xu [27]	2019	2010–2015	SEER	St. IB	41.7	23.5	265	80	185
Xu [27]	2019	2010–2015	SEER	St. IIA	75.0	0.0	113	11	102
Xu [27]	2019	2010–2015	SEER	St. IIB	26.8	16.0	599	83	516
Xu [27]	2019	2010–2015	SEER	St. III	17.9	11.8	6298	145	6153
Yin [23]	2018	2010–2015	SC	St. II + III, PM	31.7	20.0	140	70 ^§+P^	70 ^^P^
Zhong [29]	2020	2011–2018	SC	St. I–III	28.0	16.7	152	50 ^§+^	102 ^°^
*Jin* [30]	*2018*	*2004–2013*	*SEER*	*St. I + II*	*42.7*	*25.9*	*1186*	*154* ^%^	*1032* ^°-^
*Lin* [31]	*2020*	*2004–2014*	*SEER*	*St. IA*	*50.0*	*24.7*	*686*	*337* ^§+^	*349* ^°^
*Varlotto* [32]	*2011*	*1988–2005*	*SEER*	*St. I*	*47.4*	*17.2*	*1053*	*361* ^%^	*692* ^r^
*Wei* [33]	*2020*	*2004–2014*	*SEER*	*St. I*–*III*	*36.7*	*16.8*	*1562*	*781* ^$-^	*781* ^^^
*Weksler* [34]	*2012*	*1988–2007*	*SEER*	*St. I + II*	*26.9*	*7.0*	*3566*	*895* ^%^	*2671* ^r^
*Yang* [35]	*2018*	*2003–2011*	*NCDB*	*St. I*	*48.1*	*28.3*	*2301*	*681* ^§+^	*1620* ^°^

The summary of each original study includes the year of publication, period of patient recruitment, data origin, and a comment on the details of each original study, including the stage analyzed, 5-year survival rates in percentage, and the number of patients in each treatment group. Note: Studies in the lower part of the table, displayed in *italics*, were excluded from this meta-analysis due to overlapping patient cohorts from similar data sources. Abbreviations: 5-y surv.: 5-year survival; CR: Cancer Registry of Norway; NCDB: National Cancer Database; NCDR: National Cancer Data Repository; NS: ‘non-surgery group’; PM: pair-match analysis; SC: single center; SEER: Surveillance, Epidemiology, and End Results database; St.: stage analyzed; Surg.: ‘surgery group’. Footnotes: §P: surgery (S) + chemotherapy (C) ± prophylactic cranial irradiation (PCI); §: S + C; $-: S ± C ± radiation (R); $+: S + C + R; 0: S; §+: S + C ± R; %: S ± R; %+: S + R ± C; §+P: S + C ± R ± PCI; ^: C ± R; °P: C + R ± PCI; n: no information; ^P: C ± R ± PCI; °: C + R; °-: C or R; r: R.

**Table 2:** Baseline characteristics.

Number Patients	Mean Age (Years)	*p*-Value	Male (%)	*p*-Value
All patients (95,323)	64.2 ± 4.7		57.4 ± 15.2	
Surg. Group (3569)	65.1 ± 3.6	0.456	59.2 ± 17.7	0.378
Non-surg. Group (91,754)	63.3 ± 5.8		55.6 ± 12.7	

Summary of patients’ baseline characteristics, including number of patients in each group, mean age in years, and gender distribution in male %. Abbreviations: Non-surg.: ‘non-surgery group’; Surg.: ‘surgery group’.

**Table 3:** Results of the meta-analysis.

Stage	Group	5-Year Survival		ARR (%)	RRR (%)	NNT
		(%)	*p*-Value			
I–III	Surgery	39.6 ± 15.3	<0.0001	22.9 ± 17.2	26.7 ± 16.9	6.6
	Non-surgery	16.7 ± 12.7				
II + III	Surgery	36.3 ± 20.2	0.043	16.1 ± 22.9	18.3 ± 22.4	19.2
	Non-surgery	20.2 ± 17.0				

Summary of meta-analysis, including 5-year survival rates in percentage for stages I–III and combined stages II and III. Abbreviations: ARR: absolute risk reduction in percentage; NNT: number needed to treat; RRR: relative risk reduction in percentage.
